# Effects of Blood Transfusion on Exercise Capacity in Thalassemia Major Patients

**DOI:** 10.1371/journal.pone.0127553

**Published:** 2015-05-26

**Authors:** Daniela Benedetto, Carmelo Massimo Rao, Claudia Cefalù, Demetrio Oreste Aguglia, Gaia Cattadori, Domenico Giuseppe D’Ascola, Frank Antonio Benedetto, Piergiuseppe Agostoni, Susanna Sciomer

**Affiliations:** 1 Centro Cardiologico Monzino, IRCCS, Milano, Italy; 2 Azienda Bianchi Melacrino Morelli, Cardiology Department, Reggio di Calabria, Italy; 3 Azienda Bianchi Melacrino Morelli, Hematology Department, Reggio di Calabria, Italy; 4 Dipartimento di Scienze Cliniche e di Comunità, Sezione cardiovascolare, Università di Milano, Milano, Italy; 5 Dipartimento di Scienze Cardiovascolari e Respiratorie, Università “Sapienza”, Roma, Italy; Fondazione G. Monasterio, ITALY

## Abstract

Anemia has an important role in exercise performance. However, the direct link between rapid changes of hemoglobin and exercise performance is still unknown.To find out more on this topic, we studied 18 beta-thalassemia major patients free of relevant cardiac dysfunction (age 33.5±7.2 years,males = 10). Patients performed a maximal cardiopulmolmonary exercise test (cycloergometer, personalized ramp protocol, breath-by-breath measurements of expired gases) before and the day after blood transfusion (500 cc of red cell concentrates). After blood transfusion, hemoglobin increased from 10.5±0.8 g/dL to 12.1±1.2 (p<0.001), peak VO_2_ from 1408 to 1546mL/min (p<0.05), and VO_2_ at anaerobic threshold from 965 to 1024mL/min (p<0.05). No major changes were observed as regards heart and respiratory rates either at peak exercise or at anaerobic threshold. Similarly, no relevant changes were observed in ventilation efficiency, as evaluated by the ventilation vs. carbon dioxide production relationship, or in O_2_ delivery to the periphery as analyzed by the VO_2_ vs. workload relationship. The relationship between hemoglobin and VO_2_ changes showed, for each g/dL of hemoglobin increase, a VO_2_ increase = 82.5 mL/min and 35 mL/min, at peak exercise and at anaerobic threshold, respectively. In beta-thalassemia major patients, an acute albeit partial anemia correction by blood transfusion determinates a relevant increase of exercise performance, observed both at peak exercise and at anaerobic threshold.

## Introduction

Circulating hemoglobin (Hb) and exercise performance are strictly linked. Indeed, a high Hb level is considered a must for elite athletes just as anemia correction is a must in several chronic diseases such as heart failure, thalassemia, renal insufficiency, hematopoietic disorders, just to mention the most frequent ones where anemia has a pivotal role in patients’ well-being and often in their prognosis [1–3]. In the clinical field, the gold standard for exercise evaluation is peak exercise oxygen uptake (peak VO_2_), that is peak exercise cardiac output x artero-venous O_2_ content difference. The latter is Hb x O_2_sat in the systemic artery—Hb x O_2_sat in the central vein. However, no clear-cut data are presently available to define the quantitative role of Hb change in exercise performance in chronic diseases. Indeed, anemia correction, for instance in chronic kidney disease and heart failure, is usually progressive and obtained by iron and/or erythropoietin supplementation [4–7]. Accordingly, over time, anemia correction improves exercise performance by at least two mechanisms: a direct action on blood Hb level, and an indirect action related to a training effect by physical activity which progressively increases over time as anemia severity decreases. However, measurements showing the isolated effect of anemia correction in chronic diseases are presently lacking. We have previously indirectly and semi-quantitatively assessed this issue by analyzing the correlation between circulating Hb and peak VO_2_in a large population of heart failure patients [2]. In this specific population, we showed that, for each gram of Hb increase, VO_2_ increase was 109 mL/min at peak and 74 mL/min at the anaerobic threshold (AT). Thalassemia patients have a reduced exercise capacity which is due, as well as to anemia, to several causes including physical deconditioning, low rate of high energy phosphate production and utilization, and reduced oxidative capacity of myocytes [8, 9]. Moreover, a cardiogenic cause of exercise limitation is also frequently observed, being cardiac involvement frequently observed in thalassemia [10–13]. Indeed, on top of iron overload, also myocarditis, pulmonary hypertension and diabetes frequently affect cardiac function in beta-thalassemia major (TM) patients [14–16]. TM patients are a unique population. Indeed, they regularly undergo blood transfusion, so that the direct influence of Hb increase on exercise performance can easily be assessed. This study was performed to assess the role of Hb increase by hemotransfusion on VO_2_ both at peak exercise and at AT in TM patients.

## Methods

We studied 18 consecutive TM patients in stable clinical condition. Anthropometric data are reported in Table 1. Eight patients had previously undergone splenectomy and were treated with low-dose aspirin. Seven patients received a supplement of thyroid hormone. Patients were regularly followed at the Hematology Centre of Hospital E. Morelli, Reggio Calabria, Italy. Only one patient was an active smoker and showed increased myocardial iron at nuclear magnetic resonance. Renal function was normal in all but two subjects, who showed minor renal insufficiency (eGFR 90–60 mL/min). Moderate left ventricular hypertrophy was observed in one case. Three patients were diabetic and one was hypertensive on beta-blocker treatment. Patients were instructed to avoid any special activity and to maintain their usual diet and drinking habits. Patient assessment included clinical evaluation, laboratory tests, echocardiography and cardiopulmonary exercise test.

**Table 1:** Population characteristics.

	n	Mean
**AGE (years)**		33.50 ± 7.16
**GENDER (n)**	MEN 10	
	WOMEN 8	
**WEIGHT (kg)**		62.3 ± 12.2
**BMI (kg/m^2^)**		23.39 ± 4.18

BMI = Body mass index.

This report is based on the results of cardiopulmonary exercise tests performed immediately before and the day after blood transfusion. Cardiopulmonary exercise test (CPET) was carried out on a cycloergometer (Sensor Medics, V-max 400, Yorba Linda, CA) with a personalized ramp protocol aimed at obtaining peak exercise in 8 to 12 minutes [9]. Exercise protocol was the same for each patient before and after blood transfusion. All subjects had done at least one CPET before the study; we considered the previous ones as familiarization tests, and we used them to better select ramp protocols in each individual. Patients were instructed to pedal at the speed of 60 revolutions per minute. Effort was self-terminated by the subjects when they claimed that they had reached maximal effort, regardless of the respiratory quotient reached. During the exercise test, twelve-lead ECG, blood pressure, and arterial oxygen saturation were monitored. VO_2_, ventilation (VE) and carbon dioxide production (VCO_2_) were measured breath by breath. VO_2_, VE and VCO_2_ data are reported as 20-second average. AT was measured by V-slope analysis of VO_2_ and VCO_2_, and it was confirmed analyzing the ventilatory equivalents and the end-tidal pressures of CO_2_ and O_2_. Linear regression was applied to the VO_2_/work relationship measured throughout the exercise, while the VE/VCO_2_ relationship was calculated from one minute after the beginning of loaded pedaling to the end of the isocapnic buffering period [17]. Predicted peak VO_2_ values were calculated according to Hansen-Wasserman equations [18]. Cardiopulmonary exercise tests were performed in the morning immediately before and the day after blood transfusion (two units of red blood cells = 500 mL).

### Statistical analysis

Data are reported as mean ± standard deviation. Differences between pre and post hemotransfusion data were analyzed by paired t-test.

The study was part of the routine activity, and data analysis has been approved by the Institutional Review Board of Ospedale Morelli (Reggio Calabria, Italy). All patients signed a written informed consent.

## Results

**Fig 1:**
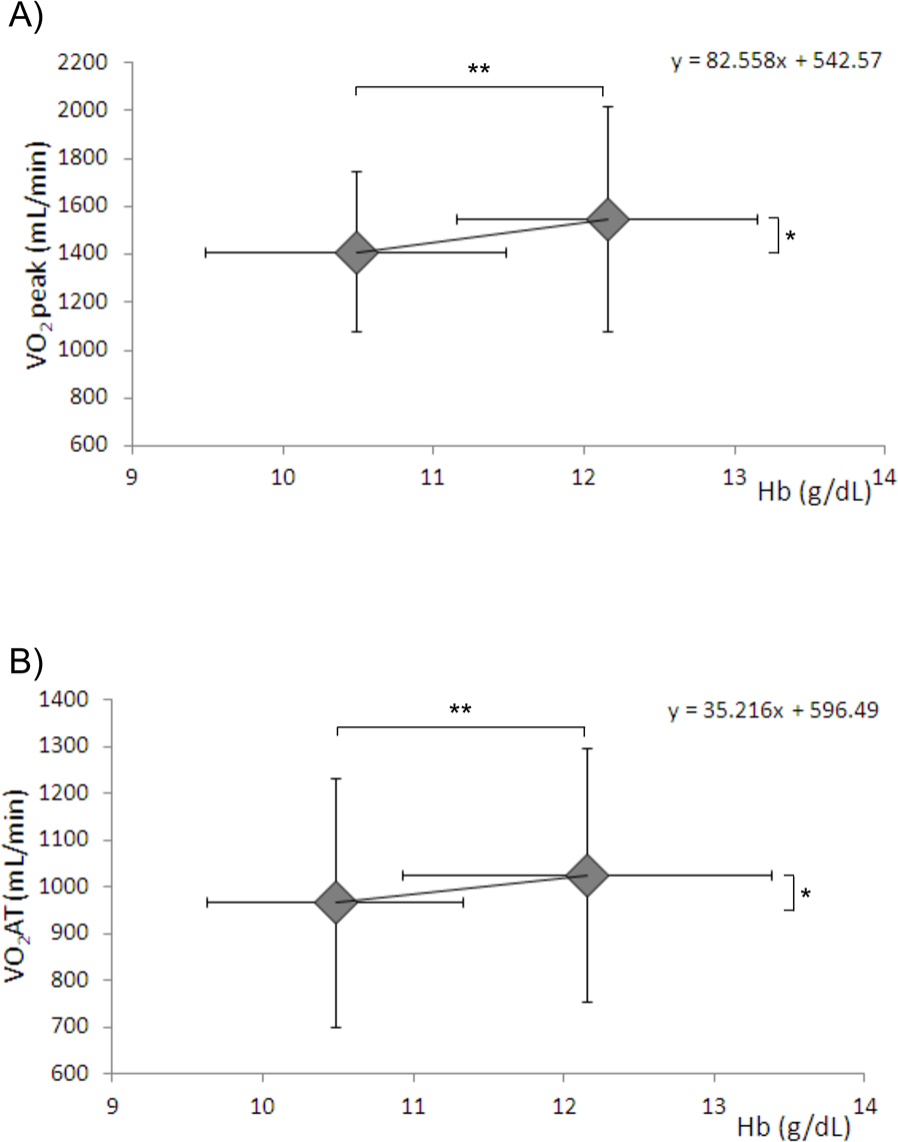
Effect of transfusion on VO_2_ and hemoglobin. VO_**2**_ and hemoglobin blood levels before and after blood transfusion at peak exercise (panel A) and at the anaerobic threshold (panel B). * = p <0.05 ** = p<0.001. Hb = hemoglobin, VO_**2**_ peak = oxygen uptake at peak exercise, VO_**2**_AT = oxygen uptake at anaerobic threshold.

Echocardiographic data showed the absence of relevant cardiac involvement, being ejection fraction 60.7±3.6% (range 56–68), left ventricle end systolic volume 32.7±11.1 (mL), left ventricle end diastolic volume 77.3±19.1 (mL); E/A was >1 in all but two subjects, E/E’ was 5.7±0.9 (range 5–7), and tricuspid annular plane systolic excursion was 20.9±2.7 cm (range 18–26). No relevant correlation was found between exercise performance and echo-Doppler parameters. After red blood cell transfusion, hemoglobin increased from 10.5±0.8 g/dL to 12.1±1.2 (p = <0.001). Resting heart rate was 88±10 b/min and 82±8 b/min (p = 0.027) before and the day after blood transfusion, respectively. Specifically, no subject had symptoms or ECG changes suggestive of exercise-induced myocardial ischemia. All patients performed uneventful maximal cardiopulmonary exercise tests (peak RER = 1.06±0.09 and 1.10± 0.07 p = 0.29), respectively. On the average, exercise performance was low, being it 52.1±13.0% of predicted values before blood transfusion. After transfusion, exercise performance remained low, although it increased to 57.7±14.01% (p = 0.015, 10.7% increase). Major cardiopulmonary exercise data are reported in Table 2. After blood transfusion, VO_2_ increased both at AT and at peak exercise in parallel with a workload increase. On the average, for 1 g of Hb, VO_2_ increased by 82.5mL/min and 5mL/min at peak and at AT, respectively (Fig 1).

**Table 2:** Cardiopulmonary exercise test measurements before and after blood transfusion.

	**PRE**	**POST**	**p**
**VO_2_peak (mL/min)**	1408 ±333	1546 ± 471	0.026
**VO_2_peak AT (mL/min)**	965 ± 26	1024 ± 26	0.044
**VO_2_peak/kg (mL/min/kg)**	23.1 ± 6.4	25.5 ± 7.9	0.009
**VO_2_peak/kg AT (mL/min/kg)**	15.8 ± 4.6	17.1±5.1	0.011
**VCO_2_peak (mL/min)**	1.5 ± 0.4	1.7 ±0.5	0.021
**VCO_2_peak AT (mL/min)**	0.9 ± 0.3	1.0 ±0.3	0.047
**O_2_ Pulse peak (mL/beat)**	8.9 ± 2.0	10.1 ± 2.5	0.0014
**O_2_ Pulse peak AT (mL/beat)**	7.8 ± 2.2	8.6 ± 1.7	0.0018
**Workload (watt)**	91 ± 26	101 ± 34	0.013
**Workload AT (watt)**	55 ± 23	61 ± 24	0.006
**TV peak (L)**	1.5 ±0.4	1.4 ± 0.4	0.5
**TV AT (L)**	1.1 ± 0.4	1.1 ± 0.4	0.75
**VE peak (L/min)**	40.5 ± 9.5	44.5 ± 11.7	0.013
**VE AT (L/min)**	24.5 ± 6.0	26.3 ± 6.8	0.067
**HR peak (bpm)**	159 ± 15	156 ± 20	0.158
**HR AT (bpm)**	126 ± 13	123 ± 15	0.163
**RR peak (bpm)**	30.6 ± 8.6	32.7 ± 9.7	0.021
**RR AT (bpm)**	23.7 ± 8.0	24.6 ±7.9	0.208
**VO_2_/work (mL/min/watt)**	10.4 ± 1.2	10.7 ± 1.2	0.286
**VE/VCO_2_slope**	23.7 ± 3.8	22.8 ± 4.2	0.287

VO_2_ = Oxygen uptake, AT = anaerobic threshold, VCO_2_ = Carbon dioxide production, TV = tidal Volume, VE = Ventilation, HR = heart rate, RR = respiratory rate.

## Discussion

This study shows that acute partial anemia correction by blood transfusion in TM patients is associated to a relevant increase of exercise performance, as evaluated both at peak exercise and at AT.

The population we studied is composed by young adults with TM. Their exercise performance is severely impaired as in similar populations previously studied [8, 9, 19]. Several are the causes of exercise limitation in TM patients on top of anemia itself, including cardiac function, sedentary attitude and muscle deconditioning, and likely a not yet completely defined form of myopathy [8]. No major cardiac involvement was evident in this population, likely due to appropriate TM treatment and regular follow-up of patients [20]. Indeed, echocardiographic reports showed normal left ventricular systolic function and volumes and, in the majority of cases, normal diastolic function. A relevant deconditioning is also likely. Indeed, because they are considered as diseased subjects, TM patients are told since childhood to avoid exercise, by family members and even by medical personnel, although there is no indication in favor of this suggestion. Indeed, none of our patients had been involved in a physical rehabilitation program, nor performed regular physical activity. As a matter of fact, we previously showed that the few thalassemia patients who perform regular physical activity do not show muscular deconditioning, which was present in patients with a more sedentary lifestyle [9].

Consequence of the deconditioning and of the scarce habit to exercise of some subjects is the RER value we recorded at peak exercise. CPET was always interrupted by the patients when they claimed that they had performed a maximal effort, and never by the medical personnel. Consequently, the average RER value, both in the first and the second test, suggests that the effort was maximal or nearly maximal from a metabolic point of view [21]. However, the huge standard deviation also suggests that this was not the case in some subjects—and likely in those subjects with a less active lifestyle.

Peak exercise improved in TM patients after blood transfusion. However, peak exercise data are dependent on patients’ effort, and one may speculate that, after blood transfusion—which is impossible to blind—patients are more confident with the effort, and that they consequently exercise more strenuously. Indeed, albeit not statistically significant, RER was higher in the post-transfusion test than in the pre-transfusion test. Notably, exercise improvement after blood transfusion was observed both at peak exercise and at AT, the latter being effort independent. Moreover, AT represents a more everyday life effort, suggesting that blood transfusion in TM patients does not only improve peak exercise performance, but also daily activity.

We have previously shown that, in a chronic heart failure population with reduced exercise capacity, the correlation between VO_2_ and Hb suggests a VO_2_ increase of 109mL/min and 74 mL/min for each gram of Hb at peak exercise and at AT, respectively. These values were obtained considering a huge population, but other variables related to peak VO_2_, such as cardiac output and O_2_ extraction, were not considered [22]. Few other studies in heart failure provided different peak VO_2_ changes for each gram of Hb increase [4–7]. However, in these studies, Hb increase was obtained by iron and/or erythropoietin treatment, which implies a prolonged treatment period during which a slow, progressive Hb increase is achieved. Consequently, as Hb increases, patients’ well-being increases, with the likely possibility of a training effect. In the present study, we observed a lower increase of VO_2_ for each gram of Hb in TM patients, specifically 82.5 and 35 mL/min/g at peak and at AT, respectively. Our study, albeit apparently simplistic and, at a first glance, even trivial, is unique, because Hb increase was acute so that cardiac output and O_2_ extraction changes are unlikely just as a training effect is. The observation at AT is particularly relevant. Indeed, up to AT, body metabolism is mainly aerobic and blood flow independent, while above AT an anaerobic component is added to the aerobic one and a non–blood-flow-dependent component of ATP production develops [17, 23]. Therefore, the fact that AT is postponed and has a significantly higher VO_2_ is an uncontaminated index of anemia correction on exercise performance. However, 35 mL/min/g is the amount of VO_2_ increase calculated at AT per gram of Hb increase; this is likely the true VO_2_ increase for each gram of Hb change. Indeed, the extent of VO_2_ increase at peak exercise, 82.5 mL/min/g, is amplified by the effects of anemia correction on exercise myocardial function and therefore on cardiac output.

Few study limitations should be acknowledged. Firstly, whenever an intervention is evaluated, a correct analysis implies that some patients are studied without the intervention (sham patients) and some other with the active intervention, or that the time course of the observation is variable. For logistic reasons, we limited our evaluation to a before and after intervention analysis. Secondly, the effects of blood transfusion on exercise performance should be considered only within the Hb values we observed and cannot be extrapolated to higher or lower Hb blood values. Thirdly, blood transfusion implies an increase of red blood cells but also an increase in intravascular volume. The latter may influence exercise hemodynamics and therefore change peak VO_2_ independently of Hb changes. No data are available in this regard in TM patients. However, rapid saline infusion is associated with a reduction of peak VO_2_ in healthy individuals [24, 25] and in chronic heart failure patients [26], suggesting that the effects on exercise performance are mainly related to Hb changes and not to intravascular volume changes. Fourthly, Hb increases during exercise in all subjects, including TM patients. However, the exercise-induced hemoconcentration is blunted in thalassemia patients, and it is negligible in thalassemia patients who have undergone splenectomy [9]. Indeed, we previously reported, in thalassemia intermedia patients, an exercise-induced Hb increase of 0.4±0.2 g/dL and 1.0±0.4 g/dL in patients who had and who had not undergone splenectomy, respectively [9]. Notably, 8 out of our 18 patients had previously undergone splenectomy for clinical reasons. Moreover, exercise-induced hemoconcentration only takes place with a relevant effort, as it is mainly due to intracellular lactic acid increase [22, 27]. Accordingly, data at AT are unaffected by exercise-induced hemoconcentration. Furthermore, we did not perform a peak exercise blood sample as we wanted to reduce patients’ discomfort as much as possible and to avoid any interference of blood sampling with maximal exercise performance which is a likely event in such fragile subjects. However, the lack of peak exercise Hb measurement cannot influence the pre and post blood transfusion peak VO_2_ vs Hb relationship. Finally, the number of TM patients we studied is definitely small. More cases are certainly needed, possibly with a higher Hb range, to confirm our findings.

In conclusion, we showed that blood transfusion increases exercise performance in TM patients with a VO_2_ increase for each Hb g = 82.5 mL/min and 35 mL/min, at peak exercise and at AT, respectively.

## Supporting Information

S1 Dataset Dataset and legend of all variables. (XLS)
